# Screening Accuracy of the Portuguese version of the Postpartum Depression Screening Scale-7 according to DSM-5 criteria

**DOI:** 10.1192/j.eurpsy.2022.262

**Published:** 2022-09-01

**Authors:** A.T. Pereira, J. Azevedo, M.J. Soares, C. Marques, M. Marques, M. Barros, F. Carvalho, D. Pereira, A. Macedo

**Affiliations:** 1 Coimbra Institute for Biomedical Imaging and Translational Research, -, Coimbra, Portugal; 2 Faculty of Medicine of University of Coimbra, Institute Of Psychological Medicine, Coimbra, Portugal; 3 Centro Hospitalar e Universitário de Coimbra, Department Of Psychiatry, Coimbra, Portugal; 4 State University of Southwest Bahia, Department Of Natural Sciences, Bahia, Brazil

**Keywords:** PDSS, Postpartum, perinatal mental health

## Abstract

**Introduction:**

The Portuguese shortest version of the Perinatal Depression Screening Scale/PDSS-7 proved to be valid and reliable, in Portugal and Brazil, but it is essential to analyze its operational characteristics before using it for screening purposes.

**Objectives:**

To determine PDSS-7 cut-off points and associated conditional probabilities to screen for major depression, according to the DSM-5.

**Methods:**

he pregnancy sample was composed of 259 women in the second trimester (Mean gestation weeks=17.83±4.750). The postpartum sample consisted of 241 women assessed between the 2^nd^-6^th^months postpartum(M=17.99±4.689 weeks postpartum). All women completed the PDSS-7 and were interviewed with the Diagnostic Interview for Psychological Distress(Pereira et al., 2017), a semi-structured clinical interview to assess the most prevalent psychiatric disorders in the perinatal period according to the DSM-5 criteria. MedCalc was used to perform ROC analysis.

**Results:**

During pregnancy, the major depression prevalence was of 4.6%(n=12). The cut-off point that maximizes the Youden Index(J=.98, 95%CI: .97-.99; AUC=.99; se=.004; p<.001) was of 18(95%CI:17-19), which resulted in a sensitivity of 100%(71.5%-100%), a specificity of 97.98%(95.3%-99.3%), a positive predictive value/+PP of 68.8%(48.0%-84.0%) and a negative predictive value/-PP of 100%. In the postpartum, the major depression prevalence was of 10.4%(n=25). The cut-off point(J=.79, 95%CI: .63-.82; AUC=.89; se=.036; p<.001) was of 14(95%CI: 12-16), with a sensitivity of 85.0%(69.3%-93.2%), a specificity of 85.0%(69.3%-93.2%), a +PP of 56.5%(46.1%-67.3%) and a -PP of 97.5%(94.6%-98.8%).

**Conclusions:**

The Portuguese version of PDSS-7 presents good combinations of sensitivity and specificity, being accurate and usable to screen for depression during pregnancy and in the postpartum both in research and primary health care.

**Disclosure:**

No significant relationships.

